# Biliary pseudolithiasis secondary to ceftriaxone therapy

**DOI:** 10.4103/0253-7613.66847

**Published:** 2010-06

**Authors:** Syed Ahmed Zaki, Preeti Shanbag

**Affiliations:** Department of Pediatrics, Lokmanya Tilak Municipal General Hospital, Mumbai, India

Sir,

Ceftriaxone is a third-generation cephalosporin widely used for the treatment of a variety of bacterial infections in children.[1] It has a broad-spectrum antibacterial activity with a long plasma half-life that permits reduced dosing schedules as compared to other cephalosporins and penicillins.[2] These factors have lead to its increased use in public hospitals in developing countries. As this antibiotic becomes more commonly prescribed, practitioners should be aware of the potential adverse-effects of the drug, especially if the adverse-event can lead to medically inappropriate interventions. We herein report nine cases of biliary pseudolithiasis secondary to ceftriaxone therapy seen at our institution.

There were nine children admitted between January to October 2009 who developed biliary pseudolithiasis after starting ceftriaxone therapy. The clinical and demographic characteristics of the patients are shown in Table 1. None of the patients had hemolytic anemia, renal or liver failure, cystic fibrosis or malignancy or had received total parenteral nutrition. None of them were taking other drugs known to be associated with biliary sludge and/or lithiasis. Complete blood count, reticulocyte count, serum calcium, liver and renal function tests were normal in these children. Their family and past history was unremarkable. All patients had received intravenous ceftriaxone (100 mg/kg/day) in two divided doses. Six patients developed abdominal pain within 1 week of starting ceftriaxone therapy and three patients developed abdominal pain after completion of their therapy, i.e. after 10 days. The abdominal pain was colicky, in the right hypochondriac region and without any aggravating or relieving factors. Ultrasonography of the abdomen was performed in all these symptomatic patients [Table 2]. Patients who developed biliary pseudolithiasis before completion of treatment were shifted to alternative therapy. All the patients responded within 1 week of stopping ceftriaxone therapy, with the ultrasonography showing no evidence of pseudolithiasis. Rechallenge with ceftriaxone was not carried out in our patients due to ethical constraints. This can be labelled as a type A class of adverse-effect. It can be considered as probable/likely adverse drug reaction as per causality assessment of suspected adverse drug reaction.[3]

**Table 1:** Clinical and demographic characteristics of patients

*Patient*	*Age (years)*	*Sex*	*Weight (kg)*	*Diagnosis on admission*
A	7.5	M	20	Typhoid fever
B	7.8	M	21	Typhoid fever
C	8.2	M	20	Meningitis
D	9.4	M	22.1	Typhoid fever
E	10	M	24.2	Urinary tract infection
F	10.6	F	21.2	Meningitis
G	11	F	27.8	Meningitis
H	12	F	25.6	Urinary tract infection
I	12	F	24.3	Meningitis

**Table 2:** Duration of ceftriaxone therapy and ultrasonographic findings of the patients

*Patient*	*Duration of ceftriaxone therapy*	*Abdominal pain*	*FirstUSG*	*Follow-up USG after 7 days*
A	6	On 6^th^ day of admission	1.6 * 0.8 cm gall stone	Normal
B	6	On 6^th^ day of admission	1.2 * 1 cm gall stone	Normal
C	10	On 12^th^ day of admission	1.8 * 1.6 cm gall stone	Normal
D	6	On 6^th^ day of admission	1.1 * 1.5 cm gall stone	Normal
E	5	On 5^th^ day of admission	1 * 1.9 cm gall stone	Normal
F	10	On 13^th^ day of admission	1.2 * 1.6 cm gall stone	Normal
G	10	On 11^th^ day of admission	1.3 * 0.4 cm gall stone	Normal
H	5	On 5^th^ day of admission	1.3 * 1.3 cm gall stone	Normal
I	6	On 6^th^ day of admission	1.3 * 1.4 cm gall stone	Normal

The first sonographic demonstration of precipitates forming in the gallbladder during ceftriaxone therapy was reported by Schaad *et al*. in 1986.[4] In subsequent reports, biliary sludge or biliary pseudolithiasis has frequently been reported with this antibiotic.[5] Ceftriaxone is mainly excreted in the urine and a significant proportion (40%) is also secreted in the bile and then eliminated via the gastrointestinal tract.[2] It is secreted unmetabolized into bile in concentrations 20–150-times that of serum concentrations and is further concentrated in the gall bladder as a calcium salt.[6] When the solubility of the drug exceeds, it leads to the formation of calcium–ceftriaxone salt precipitates that mimic gallstone on ultrasonography. With discontinuation of the drug, these “stones” resolve spontaneously, a phenomenon termed “reversible ceftriaxone biliary pseudolithiasis.”[7] The incidence of pseudolithiasis in children treated with ceftriaxone is up to 25%, but only a minority of these patients become symptomatic.[2] Papadopoulou *et al*. described 25% of children on high-dose ceftriaxone therapy (>100 mg/kg per day) developing pseudolithiasis, with only 4% having right-upper quadrant pain in association with these ultrasonographic changes.[5] Precipitates form after 4–22 days (mean, 9 days) of treatment and resolve within 2–63 days (mean, 15 days) after cessation of therapy.[8] Risk factors for biliary pseudolithiasis formation include age greater than 24 months, gram-negative sepsis, high doses of ceftriaxone (≥2 g/day), increased calcium secretion into bile (e.g., hypercalcemia), post surgery, decreased bile flow (e.g., fasting or total parenteral nutrition) and increased ceftriaxone excretion in bile (e.g., renal failure or long-term treatment with ceftriaxone).[2,6] Conservative management of ceftriaxone-associated “pseudolithiasis” is suggested in managing this clinical entity with serial ultrasounds until complete resolution of “stone” formation.[2]

In conclusion, patients receiving a high dose of ceftriaxone and developing colicky abdominal pain should be considered for ultrasound and a change in antibiotic if appropriate. Recognition of this complication will prevent unnecessary surgical interventions.
